# Trade‐offs between male fertility reduction and selected growth factors or the klotho response in a lipopolysaccharide-dependent mouse model

**DOI:** 10.1007/s43188-021-00098-x

**Published:** 2021-05-20

**Authors:** Przemyslaw Solek, Jennifer Mytych, Ewelina Sujkowska, Magdalena Grzegorczyk, Patrycja Jasiewicz, Magdalena Sowa-Kucma, Katarzyna Stachowicz, Marek Koziorowski, Anna Tabecka-Lonczynska

**Affiliations:** 1 Department of Biotechnology, Institute of Biology and Biotechnology, Collegium Scientarium Naturalium, University of Rzeszow, Pigonia 1, 35-310 Rzeszów, Poland; 2 Department of Human Physiology, Institute of Medical Sciences, Medical College of Rzeszow University, Kopisto 2a, 35-959, Rzeszow, Poland; 3 Department of Neurobiology, Maj Institute of Pharmacology Polish Academy of Sciences, Smetna 12, 31-343 Krakow, Poland

**Keywords:** Antidepressant-like substances, LPS, Growth factors, Klotho, Testis

## Abstract

The increasing number of depression cases leads to a greater need for new antidepressant treatment development. It is postulated that antidepressants may harm male fertility, but the cellular mechanism is still poorly understood. The role of growth factors and klotho protein in maintaining normal male reproductive function is well documented. Hence, the study aimed to investigate the effect of the antidepressant drug – imipramine (tricyclic AD), and other substances with antidepressant potential (ALS), administered in combination or in combination with LPS (an animal model of depression) on gene expression and protein synthesis of IGF-2 (insulin-like growth factor 2), TGF-β1 (transforming growth factor β1), NGF (nerve growth factor), KGF (keratinocyte growth factor) and protein synthesis of VEGF-A (vascular endothelial growth factor A), IGF-IR (insulin-like growth factor receptor 1), EGFR (epidermal growth factor receptor) and klotho in the testis of mice. Mice were injected intraperitoneally with selected ALS and LPS or 10% DMSO (controls) (n = 7/group) once a day for 14 days. Animals were decapitated and testes collected for RNA and protein purification. PCR and western blot methods were employed for the evaluation of growth factors and klotho expression. The results obtained indicated a decreased level of most of the analyzed genes and proteins, except KGF; its expression increased after treatment with MTEP and IMI administrated individually and after NS-398, and IMI in combination with LPS. Our results may suggest that the tested ALS and LPS can contribute to a reduction of male fertility, but NS-398, IMI, and IMI+NS-398 may also act as stimulants after LPS.

## Introduction

Depression is a multifaceted disorder with various outcomes that involve risk of serious disorders in the organism, among others. One of the most common and most active methods of depression treatment is therapy with the use of antidepressant-like substances (ALS). Unfortunately, pharmacological treatment often brings negative side effects. Therefore, it seems appropriate to search for substances with different mechanisms of action and for a different combination of drugs that will show high efficiency but will also be characterized by low toxicity and minimum side effects [1]. Similarly, therapy can have negative effects leading to reproductive disorders that result in infertility. Animal models indicate that the antidepressants used could harm fertility in both females [2] and males [3, 4]. Additionally, by modulating the activity of neurotransmitters, antidepressants may negatively affect reproductive functions in males and females [5]. At the same time growth factors have a significant impact on the proper functioning of the male reproductive system. The more detailed functions of selected factors are presented below. Vascular endothelial growth factor (VEGF) is an important factor in the development and homeostasis of reproductive, Sertoli, and Leydig cells [6]. It has also been observed that abnormal levels of VEGF can be a key factor in the pathogenesis of infertility [7]. Iinsulin-like growth factor (IGF) and insulin-like growth factor receptor (IGF-IR) stimulate steroidogenesis by increasing the density of gonadotropin receptors (LH/hCG) and the expression of key steroidogenic enzymes [8]. It was also found to reduce physiology and sperm activity as confirmed by the presence of IGF in animal and human semen [9]. Transforming growth factor (TGF) affects the process of spermatogenesis and regulates the secretion of Leydig and Sertoli cells [10]. In turn, nerve growth factor (NGF) is an important factor in the early stages of germ cell maturation, but it is also necessary for the survival of the spermatozoa by performing an anti-neurotrophic function during maturation and sperm motility [11]. The role of keratinocyte growth factor (KGF) is related to the growth and differentiation of cells in the testis [12]. Klotho is an important protein mainly associated with the ageing process. It has been shown that its deficiency causes premature ageing, neurodegeneration, pulmonary emphysema, soft tissue calcification, but also leads to gonadal atrophy [13, 14]. Two klotho isoforms were distinguished: transmembrane and secreted. Within the male reproductive system, they are located within mature germ cells as well as in Leydig and Sertoli cells [13, 14]. It was also found that, klotho controls the process of germ cell maturation and supervises the course of spermatogenesis [15]. The contribution of growth factors and klotho is, therefore, necessary to preserve male fertility.

Since the testis is the place where the male reproductive cells are formed and fluctuations of the investigated factors expression may affect the course of spermatogenesis, the results obtained may help to understand the mechanism that regulates reproductive processes in males and consequently how to treat male infertility. Although it is well known that the therapy using ALS helps to combat depression, their impact on the male reproductive system is poorly understood. Therefore, the objective of this study was to assess the effect of ALS and LPS (lipopolysaccharide *E. coli*) on the expression level of growth factors: *IGF-2*, *TGF-β1*, *NGF*, *KGF* and on protein synthesis of VEGF-A, IGF-IRβ, EGFR (epidermal growth factor) and klotho in the testis of the adult mice.

## Materials and methods

### Animals

The experiments were performed on groups of male mice of 8–10 weeks old (C57BL/6J) with an average weight of 20 ± 1 g. Each group consisted of seven animals kept in laboratory conditions of light and temperature (6:00–18:00; 21 ± 1 °C). Throughout all the experiments, constant and free access to water and food was provided. Procedures were performed during the light period (9:00–17:00) and according to the guidelines of the National Institutes of Health Animal Care and Use Committee and were approved by the Ethics Committee of the Maj Institute of Pharmacology, Polish Academy of Sciences in Krakow (Protocol No 178/2017).

### Drug administration

The following drugs were used: 3-[(2-methyl-1,3-tiazol-4-yl)ethynyl]-pyridine (MTEP, Tocris Cookson ltd., Bristol, UK); N-[2-(cyclohexyloxy)-4-nitrophenyl]methanesulfonamide (NS398, Abcam Biochemicals, UK); Imipramine (hydrochloride, Sigma-Aldrich, Saint Louis, MO, USA); LPS serotype 0127:B8 (Sigma-Aldrich).

The experiment contained 12 groups of mice administered ALS and ALS combined with lipopolysaccharide (LPS). The drugs were injected intraperitoneally (ip) once a day for 14 days (before 11:00 a.m.) as follows: MTEP (1 mg/kg) in 1% aqueous solution of Tween 80; NS-398 (3 mg/kg) dissolved in 10% DMSO; IMI (10 mg/kg) dissolved in distilled water. The above substances were also given in combinations and all details are presented in Table 1. The concentration of substancesand treatment schedule were determined based on literature research, which shows that this is a sufficient time to capture chronic changes. The duration of the spermatogenesis process in male mice was also taken into consideration. LPS (0.83 mg/kg), as a stressing factor and causative agent in mouse depression, was administered [16] 24 h before the last dose of tested substances to connect the last injection with a “depressive-like” faze after the LPS challenge [17]. The selected LPS dose was chosen based on previously reported studies [16, 18, 19] 0.24 h after the last administration, the animals were euthanized and the testis were collected for analyses.The samples of testicular tissues were freshly dissected, divided into fragments, and kept at − 80 °C until analysis.

**Table 1:** Scheme of dispensing pharmacological substances used for both parts of the experiment

Pharmacological substance		Dose	Abbreviation
Vehicle	10% DMSO (dimethyl sulfoxide)		Control (C)
Administrated substances	MTEP (3-((2-metylo-4-tiazolilo)etynylo)pirydyne	1 mg/kg dissolved in 1% Tween 80	MTEP
	NS-398 (N-(2-cykloheksyloksy-4-nitrofenylo)metanosulfonamid)	3 mg/kg dissolved in 10% DMSO	NS-398
	Imipramine (3-(5,6-dihydrobenzo[b][1]benzazepin-11-ylo)-N,N-dimetylopropano-1-amina)	10 mg/kg dissolved in distilled water	IMI
	MTEP + NS-398	1 mg/kg dissolved in 1% Tween 80 + 3 mg/kg dissolved in 10% DMSO	M1
	Imipramine + NS-398	10 mg/kg dissolved in distilled water + 3 mg/kg dissolved in 10% DMSO	M2
	LPS (lipopolisaccharide of *Escherichia coli*)	0.83 mg/kg LPS	Control (C)/LPS
	MTEP (3-((2-metylo-4-tiazolilo)etynylo)pirydyne + LPS	1 mg/kg dissolved in 1% Tween 80 + 0.83 mg/kg LPS	MTEP/LPS
	NS-398 (N-(2-cykloheksyloksy-4-nitrofenylo)metanosulfonamid) + LPS	3 mg/kg dissolved in 10% DMSO + 0.83 mg/kg LPS	NS-398/LPS
	Imipramine (3-(5,6-dihydrobenzo[b][1]benzazepin-11-ylo)-N,N-dimetylopropano-1-amina) + LPS	10 mg/kg dissolved in distilled water + 0.83 mg/kg LPS	IMI/LPS
	MTEP + NS-398 + LPS	1 1 mg/kg dissolved in 1% Tween 80 + 3 mg/kg dissolved in 10% DMSO + 0.83 mg/kg LPS	M1/LPS
	Imipramine + NS-398	10 mg/kg dissolved in distilled water + 3 g/kg dissolved in 10% DMSO + 0.83 5 mg/kg LPS	M2/LPS

### Total RNA isolation, reverse transcription (RT) and PCR analysis

Tissues homogenization in Fast Prep-24 (three times for 20 s) (MP Biomedicals LLC) was followed by total RNA isolation with the use of a column-based kit (A&A Biotechnology, Gdynia Poland). Attached in the kit DNAse treatment was conducted according to the manufacturer’s instruction. RNA samples were subjected to electrophoresis in 1% agarose gel to confirm the integrity of the RNA bands. The High Capacity cDNA Reverse Transcription Kit (Applied Biosystems; Foster City, USA) was used to obtain cDNA. Briefly, 1 µg of RNA diluted to 10 µl with sterile water was added to the reaction mixture containing: 2 µl of 10 × RT Buffer, 0.8 µl of 25 × dNTP Mix, 2 µl of 10 × RT Random Primers, 1 µl of Multiscribe Reverse Transcriptase, and 3.2 µl H_2_O. The cDNA synthesis was performed in a PCR Thermocycler (Biometra; Gőttingen, Germany) under the following conditions: 25 °C for 10 min, 37 °C for 120 min and 85 °C for 5 min. Specific primers were commercially synthesized based on the available literature: *ACTB, IGF-2, KGF, NGF,* and *TGF-1β* (Genomed, Warsaw, Poland)*.* The primer sequences are available in Table 2.

**Table 2:** Primer sequences for *IGF-2, KGF, NGF, TGF-β1*, and *ACTB* genes used in PCR

Target gene	Melting temperature (°C)	Primer sequence (5′–3′)
*ACTB–F* *ACTB–R*	55	GCAGGAGTACGATGAGTCCG (20nt) ACGCAGCTCAGTAACAGTCC (20nt)
*IGF-2–F* *IGF-2–R*	52	GCCCTCCTGGAGACTTACT (19nt) TGTCATGTCGGAAGAGCTTG (20nt)
*KGF–F* *KGF–R*	53	GTGGTATCTGAGGATTGATAAACG (24nt) CCACTGTCCTGATTTCCATGAT (22nt)
*NGF–F* *NGF–R*	49	CTGGGAGAGCTGAACATCAAC (21nt) CGCCTTGACGAAGGTGT (17nt)
TGF-β1–F TGF-β1–R	51	GGACACCAACTACTGCTTCA (20nt) GGTTCGTGAATCCACTTCCA (20nt)

For PCR reactions: 5 μl of 2Xpcr TaqNova-RED master mix (DNA Gdansk; Gdansk, Poland), 4 μl of primers (2 μl of each of the 1 μM forward and reverse primers) (Genomed; Warsaw, Poland) and 1 μl of cDNA (50 ng) were used. The conditions for RT-PCR were as follows: denaturing at 95 °C for 45 s, annealing at *IGF-2* (52 °C), *KGF* (53 °C), *NGF* (49 °C), *TGF-β1* (51 °C) and *ACTB* (55 °C) for 45 s, extension at 72 °C for 45 s, followed by the final extension at 72 °C for 10 min. The PCR amplification was conducted for 35 cycles and next, the PCR products were detected using 1% agarose gel electrophoresis with ethidium bromide. The calculated values of the optical density (by GelQuantNET software) were numerically expressed as the relative density. The *ACTB* gene was selected as the reference gene and all obtained results were normalised relative to it [20]. The use of *ACTB* was normalized and the transcription variable for each group of animals did not differ from each other.

### Western blot analysis

Frozen testicular tissues were homogenized with 2% SDS (Sigma, Saint Louis, MO, USA) and D lysing matrix (MP Biomedicals) using the high-speed benchtop FastPrep 24 MP homogenizer system for tree cycles of homogenization (20 s each). Then, to obtain supernatants, the homogenates were centrifuged at 15,000 g at 4 °C for 15 min. The protein concentration was measured using a BCA protein assay according to the manufacturer’s protocol (Thermo Scientific, Warsaw, Poland). For standard calibration, bovine serum albumin (BSA) was used (BioShop, Burlington, Canada). A western blot analysis was conducted following the standard protocol with some modifications. For analysis, 30 µg of total proteins were separated by 10% SDS-PAGE gel under reducing conditions and next transferred electrophoretically onto polyvinylidene difluoride membranes (Thermo Scientific, Warsaw, Poland). The membranes were then blocked with 1% BSA for growth factors and 3% BSA for klotho in TBST buffer (20 mMTris-HCl pH 7.5, 137 mMNaCl, 0.1% Tween 20) at room temperature for 1 h. Further, monoclonal mouse anti-VEGF-A (dilution 1:1000; #MA1-16626, RRID: AB_568763; Thermo Scientific, Poland), monoclonal mouse anti-EGFR primary antibody (dilution 1:2000; #05-1047, RRID:AB_1977165 Merck Millipore, USA), polyclonal rabbit anti-IGF-IRβ (dilution 1:500; #9038, RRID:AB_671793; Santa Cruz, USA), polyclonal rabbit anti-klotho (dilution 1:1000; #PA5-21078, RRID:AB_11153007; Thermo Scientific, Poland) or polyclonal rabbit anti-β-actin (dilution 1:10,000; #PA1-16889,RRID: AB_568434; Thermo Scientific, Poland) diluted in 1% BSA in TBST were applied (ON, 4ºC). Then, the membranes were washed four times for 5 min and next the horseradish peroxidase-conjugated anti-rabbit IgG (1:80,000; #A0545, RRID: AB_257896; Sigma, Saint Louis, MO, USA), or anti-mouse IgG (1:80,000; #A9044, RRID: AB_258431; Sigma, Saint Louis, MO, USA) secondary antibodies were used to detect conjugates and the antibody-antigen complexes. Additional four washings were followed by the use of an ECL detection kit (BioRad, Warsaw, Poland) and the Fusion Fx7 (Viber Lourant) system to visualize the bands on the blots. The densitometric analysis was completed with the use of GelQuantNET software and β-actin bands were used for normalization.

### Statistical analysis

Statistical analyses were conducted using GraphPadPrism 6.0 (GraphPad Software Inc., California, USA). The population distribution were normal. The results represent mean ± SD and were analysed using one-way ANOVA followed by Dunnett’s multiple comparison post hoc test in RT-PCR and western blot analyses. A probability of *p* < 0.05 was considered to be statistically significant and presented as: ∗,^, *p* < 0.05; ∗∗,^^, *p* < 0.01; ∗∗∗,^^^, *p* < 0.001.

## Results

### A antidepressant-like substances affect expression of growth factors genes in mice testicular tissues

**Fig. 1:**
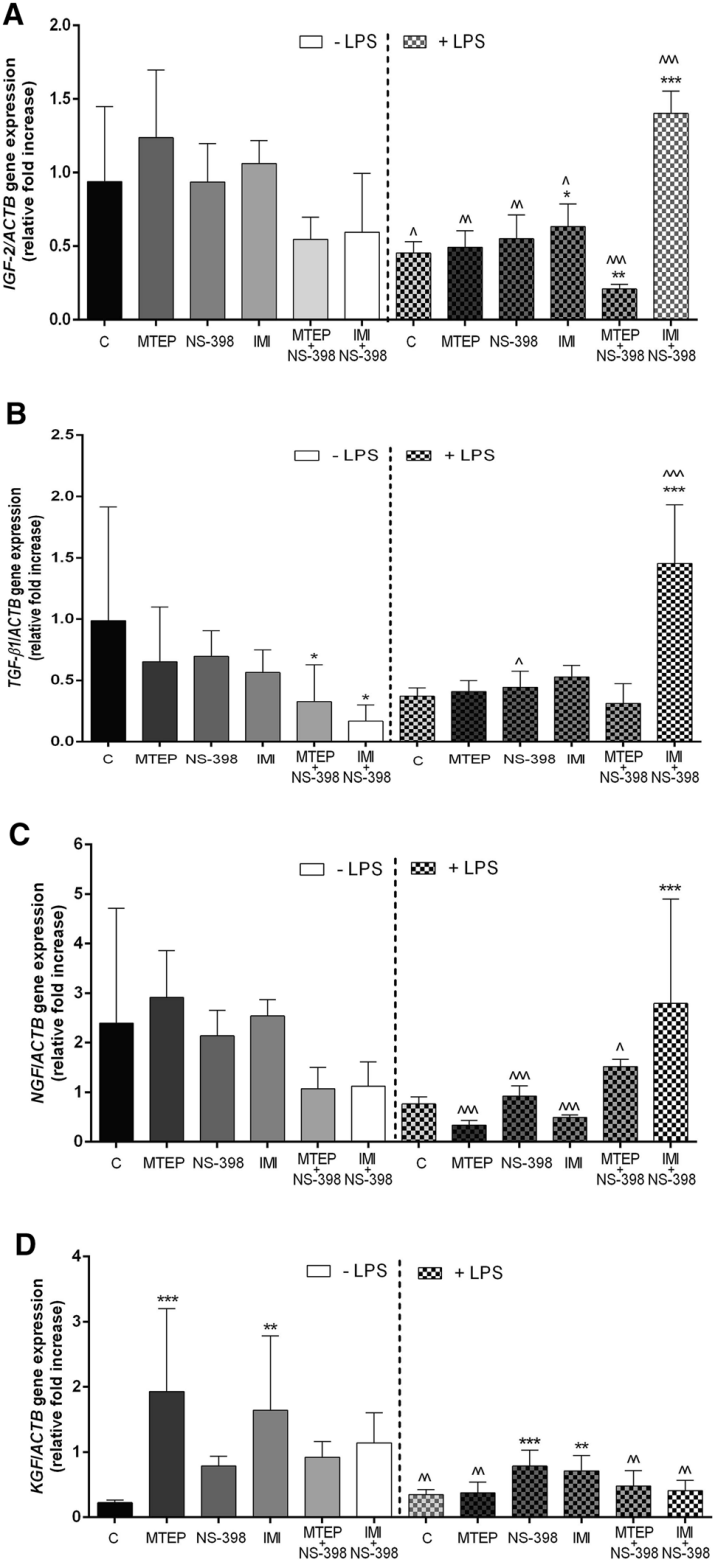
mRNA expression of: **A** *IGF-2*, **B** *TGF-β1*, **C** *NGF*, **D** *KGF* analyzed by RT PCR technique in mouse (C57BL/6J) testis; Bars indicate mean ± SD, *n* = 6, asterisks indicate comparison between control group (C) and analysed groups after administration of: MTEP, NS-398, IMI, MTEP + NS-398 (M1), IMI + NS-398 (M2) with and without LPS; ^^^,***p < 0.001, ^^,**p < 0.01, ^,*p < 0.05; (*) indicate comparison between control group and used substances, (^) indicate comparison between group after the same substances used LPS-non-treated and treated, no indication – no statistical significance (*p* > 0.05) (one –way ANOVA with Dunnett’s a posteriori test). The results were normalized to *ACTB*

**Fig. 2:**
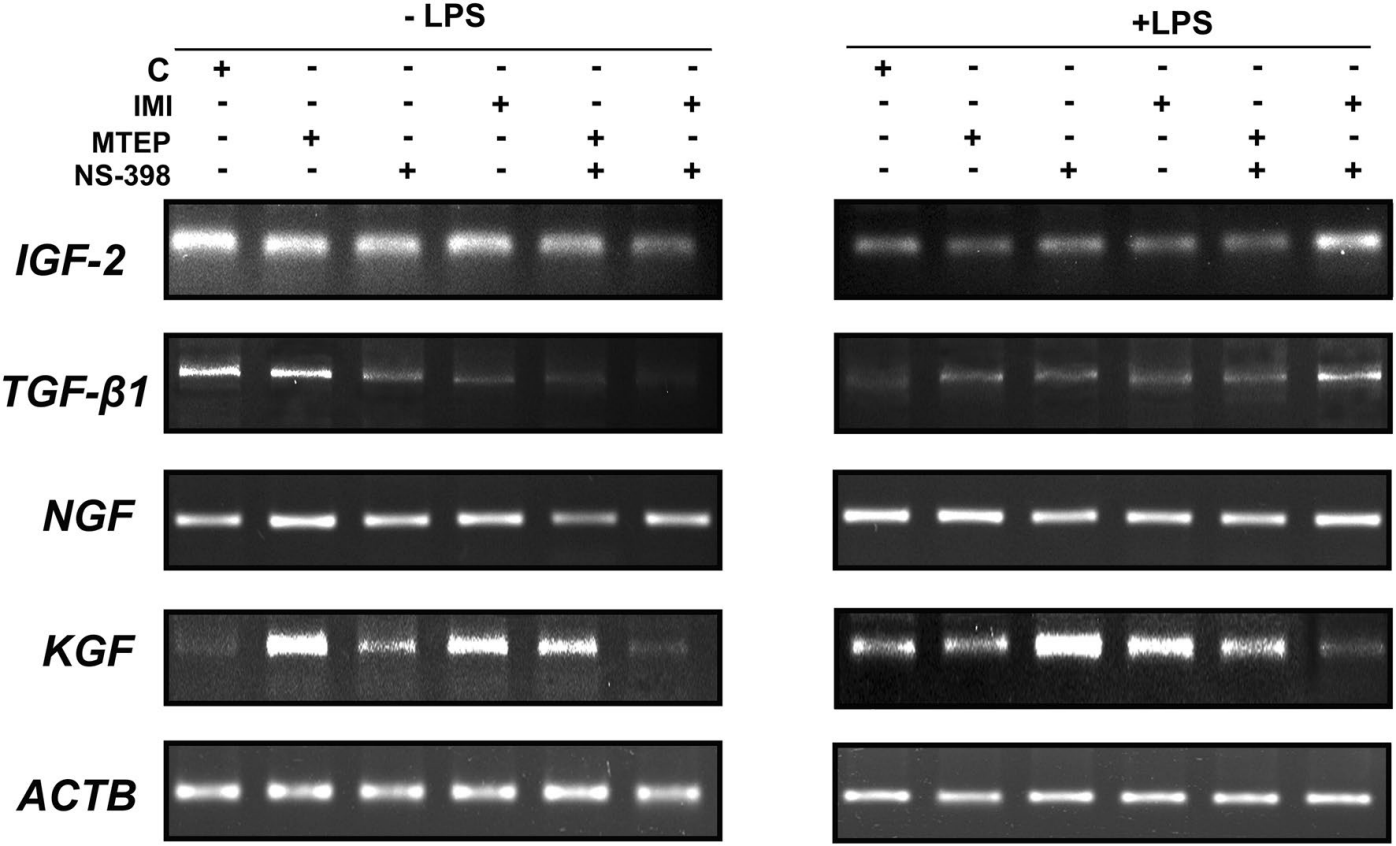
Ethidium bromide staining of the results of RT PCR for *IGF-2*, *TGF-β1*, *NGF*, *KGF* mRNA in mouse (C57BL/J) testis; representative images of gels after administration of: vehicle (C) MTEP, NS-398, IMI, MTEP + NS-398 (M1), IMI + NS-398 (M2) with and without LPS

Analysis of *IGF-2* mRNA expression in mouse testis did not reveal any differences between antidepressant-like groups and the control group (vehicle) (*p* > 0.05) (Figs. 1A, 2). After LPS administration, statistically significant differences were noted in the expression of *IGF-2* mRNA when compared to the animals without LPS. The expression increased in IMI/LPS (*p* < 0.05) and IMI + NS-398/LPS (*p* < 0.001) and the levels were as follows: 1.399-fold higher for IMI/LPS and 3.092-fold higher for IMI + NS-398/LPS versus the control group. A decrease of *IGF-2* mRNA was observed in the MTEP + NS-398/LPS group and was 2.16-fold lower than in the control group (*p* < 0.01) [F(5,36) = 75.17; *p* < (0.0001)] (Figs. 1A, 2). The *IGF-2* expression was also significantly decreased compared with almost every analysed group (except in the IMI + NS-398/LPS group) without and after the LPS challenge. The obtained level for the control group with LPS was 2.072-fold lower (*p* < 0.05), for MTEP/LPS it was 2.511-fold lower (*p* < 0.01), for NS-398/LPS it was 1.701-fold lower (*p* < 0.01) and finally for MTEP + NS-398/LPS it was 1.673-fold lower (*p* < 0.05). The exception was the IMI + NS-398/LPS group where a significant increase in the expression level of *IGF-2* mRNA was observed and it was 2.354-fold higher than in the group without LPS (*p* < 0.001) [F(11,72) = 12.72; *p* < 0.0001] (Figs. 1A, 2).

The analysis of *TGF-β1* mRNA expression in mouse testis after administration of ALS showed significantly lower levels in MTEP + NS-398 and IMI + NS-398 groups (*p* < 0.05, *p* < 0.05; respectively) and they were 3.018-fold and 5.88-fold lower versus the control group [F(5,36) = 2.827; *p* = 0.0297] (Figs. 1B, 2). When we compared the *TGF-β1* mRNA expression after LPS administration we observed a significantly higher level in the IMI + NS-398/LPS group (*p* < 0.001) (3.918-fold higher versus the control group) [F(5,36) = 26.72; *p* < 0.0001] (Figs. 1B, 2). After LPS challenge the *TGF-β1* mRNA expression significantly decreased in the relation to the same group without LPS for the NS-398/LPS group (*p* < 0.05; 0.637-fold lower level). For the IMI+NS-398/LPS group an 8.7-fold increase in the expression of *TGF-β1* versus the IMI+NS-398 group without LPS administration was observed (*p* < 0.001) [F(11,72) = 6.708; *p* < 0.0001] (Figs. 1B, 2).

The expression of *NGF* mRNA did not change after ALS administration (*p* > 0.05) (Figs. 1C, 2). After LPS challenge the *NGF* mRNA expression increased significantly for IMI + NS-398/LPS (*p* < 0.001) and the obtained level was 3.665-fold higher versus the control group [F(5,36) = 7.693; *p* < 0.0001] (Figs. 1C, 2). However, when levels of *NGF* mRNA expression were compared between the same groups without LPS and after LPS administration an 8.712-fold decrease was observed in the MTEP/LPS group (*p* < 0.001), a 2.322-fold decrease in the NS-398/LPS group (*p* < 0.001) and a 5.219-fold decrease in IMI/LPS group (*p* < 0.01). At the same time, in the MTEP + NS-398/LPS (1.415-fold higher; *p* < 0.05) and IMI + NS-398/LPS groups (2.489-fold higher; *p* < 0.01), the level of *NGF* significantly increased [F(11,72) = 6.239; *p* < 0.0001] (Figs. 1C, 2).

The mRNA expression level for *KGF* was 8.573-fold higher in the MTEP and 7.306-fold higher in the IMI groups versus the control group (*p* < 0.001, *p* < 0.01; respectively) [F(5,36) = 4.909; *p* = 0.0016] (Figs. 1D, 2). Additionally, after LPS administration, the *KGF* gene expression level was 2.873-fold higher in the NS-398/LPS group and 2.043-fold higher in the IMI/LPS group versus the control group (*p* < 0.001, *p* < 0.001; respectively) [F(55.36) = 6.312; *p* = 0003] (Figs. 1D, 2). Comparison of gene expression level for *KGF* between groups without LPS and after LPS challenge showed significant increase in the control group (1.546-fold higher; *p* < 0.01), but a decrease was observed in the MTEP/LPS group (5.13-fold lower; *p* < 0.01), MTEP + NS-398/LPS group (1.932-fold lower; *p* < 0.01) and IMI + NS-398/LPS group (2.817-fold lower; *p* < 0.01) [F(11,72) = 6.869; *p* < 0.0001] (Figs. 1D, 2).

### A antidepressant-like substances affect protein synthesis of growth factors and klotho in mice testicular tissues

**Fig. 3:**
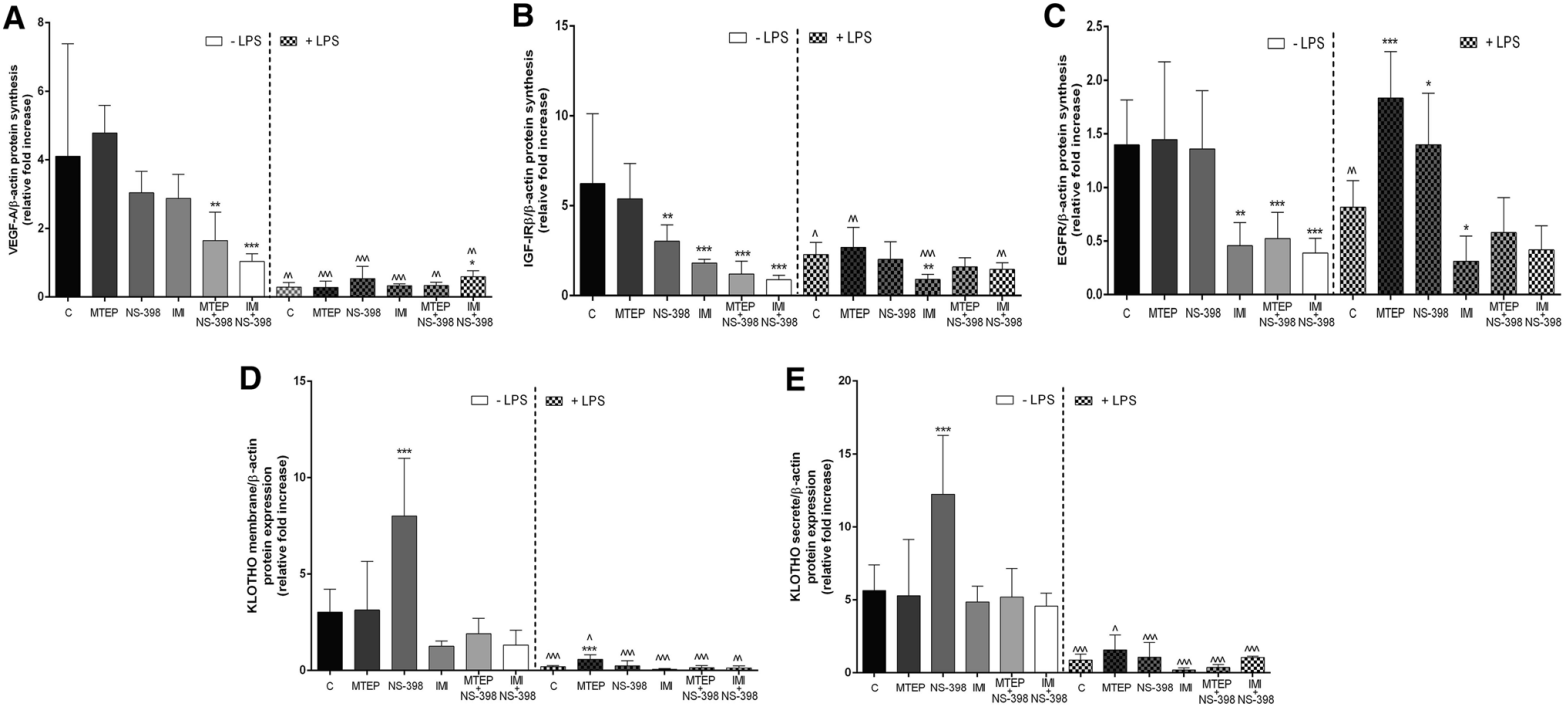
**A** VEGF-A, **B** IGF-IRβ, **C** EGFR, **D** klotho membrane isoform, **E** klotho secreted isoform protein levels analyzed by Western blot technique in mouse (C57BL/6J) testis. Bars indicate mean ± SD, *n* = 6; asterisks indicate comparison between control group and analysed groups after administration of: MTEP, NS-398, IMI, MTEP + NS-398 (M1), IMI + NS-398 (M2) with and without LPS; ^^^,***p < 0.001, ^^,**p < 0.01, ^,*p < 0.05; (*) indicate comparison between control group and used substances, (^) indicate comparison between group after the same substances used LPS-non-treated and treated; no indication – no statistical significance (*p* > 0.05) (one –way ANOVA with Dunnett’s a posteriori test). The results were normalized to β-actin

**Fig. 4:**
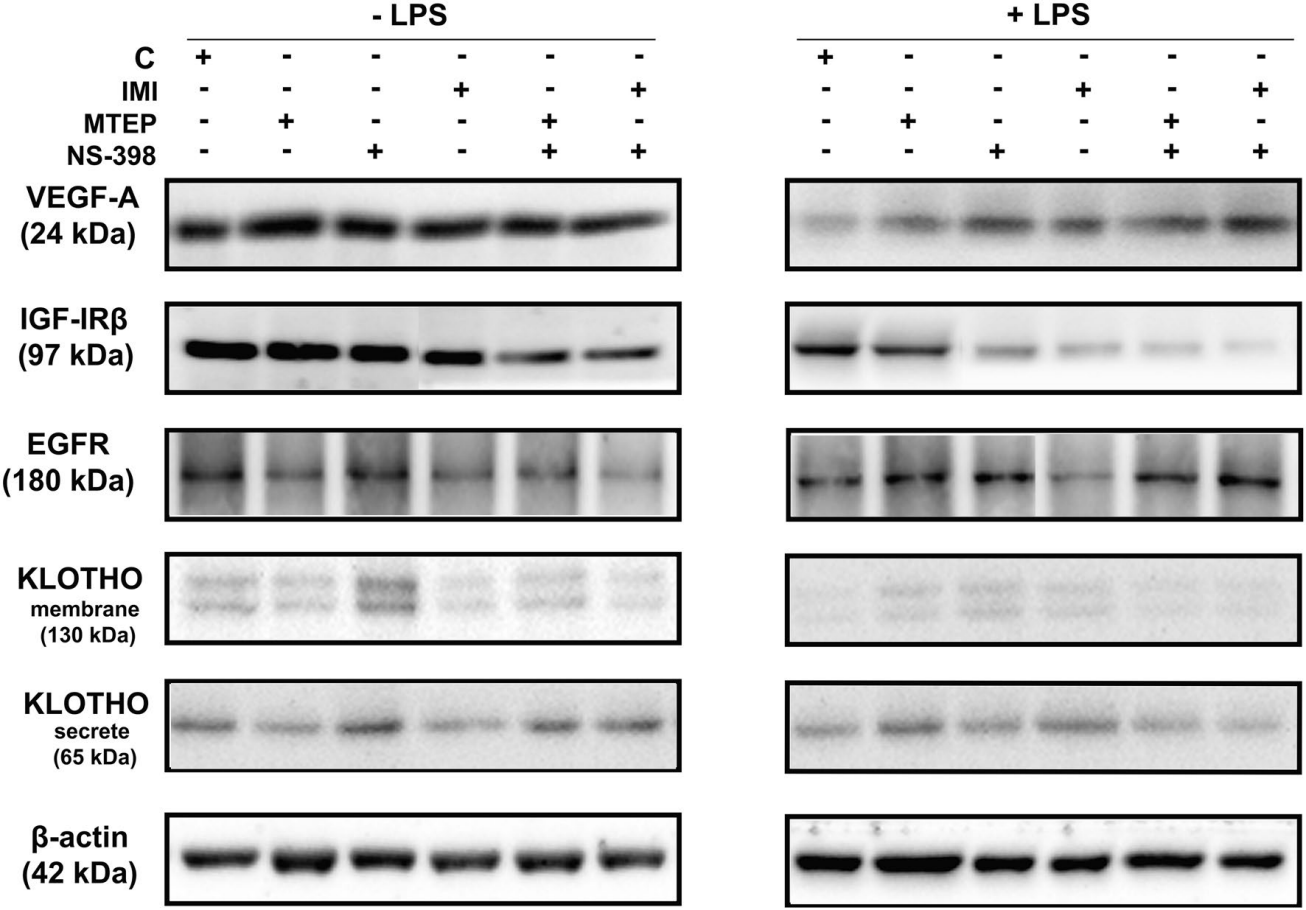
Representative immunoblots of: VEGF-A, IGF-IRβ, EGFR, KOTHO membrane isoform, KLOTHO secrete isoform and β-actin proteins in mouse (C57BL/6J) testis after administration of: vehicle (C), MTEP, NS-398, IMI, MTEP + NS-398 (M1), IMI + NS-398 (M2) with and without LPS

The measurements of VEGF-A protein level revealed a significant decrease in MTEP+NS-398 (*p* < 0.01) and IMI+NS-398 (*p* < 0.001) groups versus the control group (vehicle). Obtained levels of protein synthesis were 2.496-fold lower for MTEP + NS-398 and 3.96-fold lower for IMI+NS-398 [F(5,36) = 6.490; *p* = 0.0002] (Figs. 3A, 4). After LPS administration, only in the IMI+NS-398/LPS group was a significantly higher level of VEGF versus the control group (2.041-fold higher level; *p* < 0.05) confirmed [F(5,36) = 3.481; *p* = 0.114] (Figs. 3A, 4). Additionally, the level of VEGF-A protein synthesis after LPS challenge repeatedly decreased: for the control group/LPS (*p* < 0.01), for MTEP/LPS (*p* < 0.001), for NS-398/LPS (*p* < 0.001), for IMI/LPS (*p* < 0.001), for MTEP + NS-398/LPS (*p* < 0.01) and for IMI + NS-398/LPS (*p* < 0.01) [F(11,72) = 16.84; *p* < 0.001] (Figs. 3A, 4).

Quantitaive analysis of IGF-IRβ protein revealed that the level after NS-398 treatment was decreased by 2.059-fold (*p* < 0.01), for IMI by 3.424-fold (*p* < 0.001), for MTEP + NS-398 by 5.152-fold (*p* < 0.001) and for MTEP + NS-398 by 7.014-fold (*p* < 0.001) versus control group [F(5,36) = 10.33; *p* < 0.0001] (Figs. 3B, 4). After LPS administration only in the IMI/LPS group was the level of protein synthesis lower than in the control group. The measurement of IGF-IRβ synthesis showed a 2.027-fold decrease for IMI/LPS (*p* < 0.01) [F(5,36) = 5.480; *p* = 0.0008] (Figs. 3B, 4). However, when comparing the level of IGF-IRβ protein synthesis in individual groups of animals treated with analysed substances without LPS and after LPS challenge, a significant decrease was observed in the control/LPS group (2.795-fold lower; *p* < 0.05), in the MTEP/LPS group (twofold lower, *p* < 0.01) and in the IMI/LPS group (1.853-fold lower; *p* < 0.001) [F(11,72) = 10.37; *p* < 0.0001]. In the MTEP + NS-398/LPS group the level of IGF-IRβ protein synthesis was significantly elevated versus the MTEP + NS-398 group without LPS (1.640-fold higher; *p* < 0.01) [F(11,72) = 10.37; *p* < 0.0001] (Figs. 3B, 4).

As presented in Fig. 3C, the quantitative analysis of the EGFR protein revealed a significant decrease in IMI, MTEP + NS-398 and MTEP + NS-398 groups (*p* < 0.01, *p* < 0.01, *p* < 0.001; respectively) obtaining a level 3.061-fold, 2.669-fold and 3.616-fold lower than the control group [F(5,36) = 10.14; *p* < 0.0001] (Figs. 3C, 4). After LPS administration, a 2.246-fold higher level of EGFR protein expression in the MTEP/LPS group (*p* < 0.05) versus the control group was observed [F(5,36) = 21.86; *p* < 0.0001]. A similar increase was observed in the NS-398/LPS group (1.713-fold, *p* < 0.05) [F(5,36) = 21.86; *p* < 0.0001]. While in the IMI/LPS group the relative optical density of EGFR protein synthesis was significantly lower than in the control group, by 4.173-fold (*p* < 0.01) [F(5,36) = 21.86; *p* < 0.0001]. A significantly decreased level of EGFR protein synthesis was observed only in the control group after LPS challenge in comparison to the control group without LPS (1.71-fold lower; *p* < 0.01) [F(11,72) = 13.31; *p* < 0.0001] (Figs. 3C, 4).

The presented expression of klotho protein for both: the membrane isoform (approximately 130 kDa) and secreted isoform (approximately 65 kDa) showed similar differences in expression patterns. In the NS-398 group a significant increase for membrane isoform (2.17-fold; *p* < 0.001) [F(5,36) = 14.924; *p* < 0.0001] and for secreted isoform (2.647-fold; *p* < 0.001) [F(5,36) = 9.064; *p* < 0.0001] was observed (Figs. 3D, 4). Interestingly, only for the membrane isoform of klotho, the level of protein synthesis was significantly elevated (2.895-fold higher; *p* < 0.001) in the MTEP/LPS group versus the control/LPS group [F(5,36) = 9.414; *p* < 0.0001] (Figs. 3D, 4). No differences were obtained for the secreted isoform of klotho (*p* > 0.05) (Figs. 3E, 4). After LPS administration in all analysed groups a downregulated level of expression for both membrane and secreted form of klotho protein was observed when compared to appropriate groups without LPS [respectively: F(11,72) = 23.97; *p* < 0.0001; F(5,36) = 24.10; *p* < 0.0001] (Figs. 3D–E, 4). The decreased levels for membrane and secretedform were several times lower. For the membrane form, they were as follows: for control/LPS 15.125-fold (*p* < 0.001), for MTEP/LPS 5.411-fold (*p* < 0.001), for NS-398/LPS 34.48-fold (*p* < 0.001), for IMI/LPS 24.09-fold (*p* < 0.001), for MTEP + NS-398/LPS 13.293-fold (*p* < 0.001) and for IMI + NS-398/LPS 10.224-fold (*p* < 0.001). For the secreted form they were as follows: for control/LPS 6.512-fold (*p* < 0.001), for MTEP/LPS 3.353-fold (*p* < 0.05), for NS-398/LPS 11.559-fold (*p* < 0.001), for IMI/LPS 24.427-fold (*p* < 0.001), for MTEP + NS-398/LPS 14.163-fold (*p* < 0.001) and for IMI + NS-398/LPS 4.349-fold (*p* < 0.001) (Figs. 3D–E, 4).

**Fig. 5:**
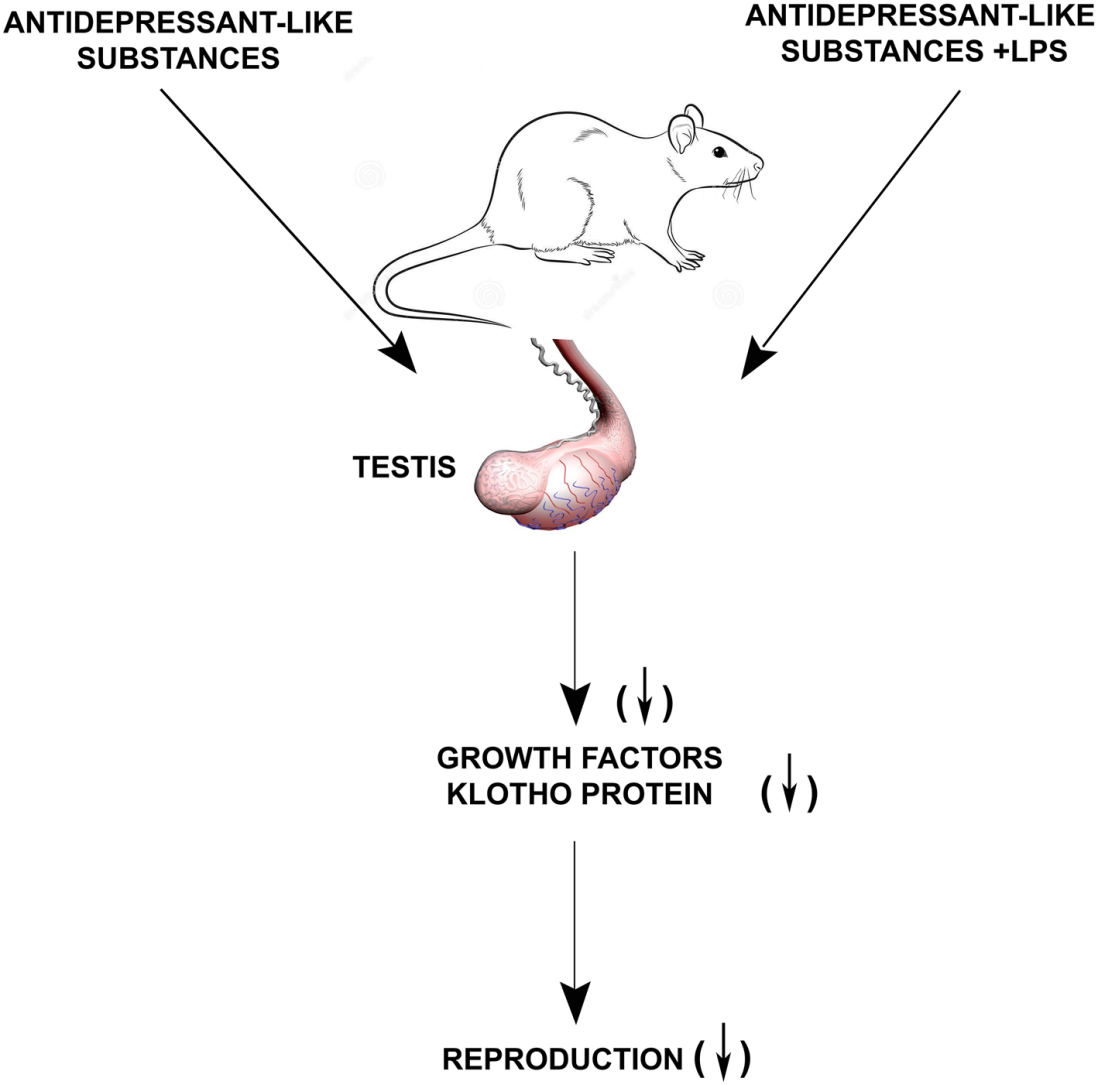
Scheme of the effect of antidepressant-like substances on mouse testis

The results obtained are presented schematically in Fig. 5.

## Discussion

The results presented in this work, allow us, for the first time to observe the effect of ALS in stress conditions (after LPS challenge), separately but also in combination, on growth factors and klotho expressionin in mouse testis. This assessment can be helpful in determining the condition of the male reproductive system in the face of an increasing number of cases of depression. Our research was aimed at finding new antidepressant therapies that will evoke the least side effects, especially in the reproductive system [21]. The combination of different substances may be associated with a synergy of action on individual structures in the organism [22]. In the available literature, results indicate a substantial regulatory contribution of growth factors and klotho to the normal functioning of the male reproductive system. These factors may be markers of testicular activity, assessed by differences in the level of gene expression (mRNA) or protein synthesis after ALS and LPS administration [23, 24]. Antidepressants are used in depression to affect neurotransmitters in the brain that determine sadness and emotions balancing. These substances belong to several groups with different mechanisms of action. Among the substances used in our study, we can distinguish: 3-[(2-methyl-1,3-tiazol-4-yl)ethynyl]-pyridine (MTEP), N-[2-(Cyclohexyloxy)-4-nitrophenyl]-methanesulfonamide (NS-398) and imipramine ((3-(5,6-dihydrobenzo[b][1]benzazepin-11-ylo)-N,N-dimetylopropan-1-amina) (IMI). Each is characterised by a different mechanism of action. MTEP is a selective group I metabotropic glutamate receptor (mGluRs; specially mGluR5) antagonist with the ability to cross the blood–brain barrier. This process also affects activation of the expression of neurotrophic factors in the brain and thus has an antidepressant-like effect [25, 26]. The presence of mGluRs was also found in the testes, which may be important for the functional role in the male reproductive system as a key element that determines mouse fertility [27]. NS-398 is a cyclooxygenase 2 inhibitor (COX-2) blocking the formation of prostaglandins [28], and based on available data, an increase of prostaglandin E2 secretion (PGE2) and COX-2 expression is observed during depression [29]. Blocking COX-2, results in inhibition of arachidonic acid (AA) synthesis, leading to disturbances in testosterone production by Leydig cells [30–32]. Furthermore, AA is an important enzyme in the regulation of gene transcription for steroidogenesis, so its deficiency may lead to disorders in the functioning of the testes [33]. COX-2 inhibition has also been shown to influence IL-1β, TNFα, and PGE, and thereby modulate clinical symptoms of depression [29]. Furthermore, our earlier research documented COX-2 participation in antidepressant-like effects observed after MTEP treatment [18]. In contrast, imipramine inhibits the reuptake of serotonin and noradrenaline in the brain and acts through the “cytosolic” phospholipase A2 (cPLA) pathway [34].

In our research, we generally observed a statistically significant decrease in gene and protein expression for growth factors and klotho. The results obtained revealed a reduced level of mRNA expression and protein synthesis after administration of LPS, which suggests a negative effect on mechanisms regulating testicular activity. After administration of ALS only, we showed a decrease of VEGF-A protein synthesis in MTEP + NS-398 and IMI + NS-398 groups. On the other hand, following LPS challenge, a significant increase in expression of VEGF-A protein was demonstrated for the combination of IMI + NS-398 which is likely to mobilise testicular cells to synthesis VEGF-A protein. However, the observed decrease in other groups may suggest a contribution of induced stress to the reduction of testicular activity, manifested by disturbances in the normal course of physiological processes in the testes. Consequently, the process of spermatogenesis and steroidogenesis may be weakened, sometimes leading to infertility[7]. Some studies demonstrate that VEGF positively affects the proliferation of spermatogenic cells or sperm motility [6, 35] and the presence of VEGF in semen contributes to the fertilization process [36–39]. VEGF is a factor in the development and homeostasis of germ cells (in seminal epithelium), Leydig and Sertoli cells [6, 40] and based on the results of Agrawal et al. (2002) abnormal levels of VEGF may have a destabilizing effect on the testicular cells and be a key factor in the pathogenesis of infertility [7].

The IGFβ receptor (IGF-IR β), is necessary to maintain the activity of insulin-like growth factor [41] and in our study, the level of protein expression was decreased in almost all tested groups. Only in the NS-398/LPS and MTEP + NS-398/LPS groups was a decrease not observed. In contrast, gene expression for *IGF-2* in the group without LPS did not show any significant changes, but after LPS challenge, for both, IGF-IRβ protein synthesis and *IGF-2* gene expression in IMI + NS-398/LPS groups, the levels were significantly elevated. This increase may provide a protective function of imipramine with NS-398 combination under stress conditions. The available literature indicates that IGF is responsible for the stimulation of steroidogenesis, increasing the density of gonadotropin receptors (LH/hCG), and the expression of key steroidogenic enzymes [42–44]. The applied therapy may therefore result in a limitation of the steroidogenesis process. In addition, IGF is an important regulator of testicular function [44–47], and it’s deficiency may contribute to male fertility disorders [9, 48].

The regulatory role of the next growth factor analysed, EGF and EGFR on testicular function of different animals has been verified [24, 49–51]. Several studies indicate local regulatory functions of EGF on reproductive organs such as DNA synthesis and steroidogenesis in Leydig cells [52]. In our study, we observed downregulated level of EGFR protein expression for IMI and MTEP + NS-398 groups. Additionally, EGFR protein synthesis decreased for IMI/LPS, as a typical antidepressant drug. Furthermore, we observed an increase in MTEP/LPS and NS-398/LPS groups which may protect testicular cells against stress conditions.

Klotho protein is a very important factor in the control of spermatogenesis and its presence was previously determined in mouse testis [13]. A lack of klotho causes atrophy of the seminiferous tubules and stops spermatogenesis [13] and its decrease negatively affects the maturation of the reproductive cells and steroidogenesis [53]. This factor can determine both, the quality and quantity of sperm [54–57]. In our study, the upregulated klotho protein expression was observed after NS-398 administration for both membrane and secreted form and in the MTEP/LPS group for the membrane form. Such an increase can have a mobilising effect on testicular function. However, after LPS challenge, in all studied groups, the level of klotho expression decreased compared to groups without LPS. It seems very likely that all tested substances may negatively affect the function of the testis.

Interestingly, in the case of *KGF* analysis, the application of MTEP and imipramine, increased gene expression level. Similarly, after LPS with NS-398 and IMI, an increase in *KGF* gene expressionwas found. After LPS only, we obtained elevated *KGF* gene expression, while in MTEP/LPS, MTEP + NS-398/LPS, and IMI + NS-398/LPS groups the level of expression was reduced. The results obtained may indicate a very individual effect of ALS and LPS on *KGF* expression. It seems, therefore, that the substances used may have a selectively positive effect on the reproductive system, through *KGF*, as its presence has been found previously especially in Leydig cells [58]. The wide range of *KGF* activity is also related to the regulation of testicular function by affecting steroidogenesis, epithelial, Sertoli, and Leydig cell development [58].

The observed decrease of *NGF* gene expression in MTEP/LPS, NS-398/LPS, and MTEP + NS-398/LPS groups after LPS may indicate the contribution of LPS administration that imitates inflammation. *NGF* is an immunomodulator by regulating the secretion of mast cells, whose task is to recognize the antigen as well as the release of cytokines and other substances to fight the unwanted state of the body [59, 60]. Furthermore, *NGF* present in Sertoli and Leydig cells affects the secretion of androgens and the course and regulation of the spermatogenesis process [61, 62]. The above information demonstrates that MTEP therapy in the presence of inflammation may negatively affect the functioning of the reproductive system. The IMI+NS-398/LPS group increase of *NGF* gene expression obtained in this study suggests that these substances may cause a good mobilising effect on testicular cells under stress conditions.

The TGF-β1 factor affects the production of testosterone during steroidogenesis, and together with other factors such as EGF, may determine other functions in the testis [63]. In this study, following application of MTEP+NS-398 and IMI+NS-398, *TGF-β1* expression decreased. Increased *TGFβ1* gene expression was demonstrated only in the IMI+NS-398/LPS group, and similar to the case of *IGF-2* and *NGF* level of expression, we suppose that imipramine together with NS-398 may act as a protector against stress condition after LPS challenge.

The frequently observed decrease in the level of expression of the studied genes and proteins after the use of MTEP and NS-398 alone and in combinations may be explained by their way of action, which together can give additional synergy effects.

The results obtained in our experiments, in most cases, seem to indicate negative effects of the use of individual ALS on the testis. These effects may result from the toxic action, but they may also be a result of the association of certain substances and LPS. In summary, the results presented in this paper may suggest that ALS given in various combinations, can significantly affect the functioning of the male reproductive system by changing the levels of growth factors and klotho. It is also possible that these substances may carry the risk of disturbing the course of normal physiological processes in mouse testes, which may determine their reproductive function. However, the above suggestions require further detailed research.
